# Importance of the Mean Rate of Pressure Change of the Pulmonary Artery (dP/dt mean PA) in Patients with Pulmonary Arterial Hypertension

**DOI:** 10.1055/s-0043-1769932

**Published:** 2023-06-20

**Authors:** Theodoros Sinanis, Alexander Schmeißer

**Affiliations:** 1 Department of Cardiology, Heart Center Duisburg, Duisburg, Germany; 2 Department of Cardiology, University Clinic Magdeburg, Magdeburg, Germany

**Keywords:** pulmonary hypertension, 6-minute walk test, pulmonary artery

## Abstract

**Background** Pulmonary arterial hypertension (PAH) is a rare disease of cardiopulmonary circulation characterized by elevated pressure in the pulmonary artery. The right-heart catheter is the gold standard for diagnosis, but there is interest in identifying additional prognostic indicators. The aim of this study was to examine the importance of the rate of pressure change of the pulmonary artery (dP/dt mean PA) in patients with PAH.

**Methods** We retrospectively analyzed data from 142 patients with PAH (exclusively clinical group 1) and examined the statistical correlation of dP/dt mean PA with vascular, right ventricular, and clinical parameters. Data was collected mostly from the right heart catheterization and the transthoracal echocardiography at presentation.

**Results** dP/dt mean PA showed a significant correlation with systolic pressure of the pulmonary artery ( *n* = 142, *R* ^2^ = 56%, *p* < 0.001), pulmonary vascular resistance ( *n* = 142, *R* ^2^ = 51%, *p* < 0.001), the rate of pressure change in the right ventricle ( *n* = 142, *R* ^2^ = 53%, *p* < 0.001), and the right ventricular fractional area change ( *n* = 110, *R* ^2^ = 51%, *p* < 0.001). Receiver operating characteristic curve analysis showed that dP/dt mean PA had the highest prognostic value in predicting increase in the 6-minute walk test and decrease in the N-terminal-probrain natriuretic peptide after the initiation of PAH therapy, with an area under the curve of 0.73.

**Conclusion** Our findings suggest that dP/dt mean PA may be a useful prognostic indicator in the treatment of patients with PAH, and further research is warranted to validate this parameter.

## Introduction

Pulmonary hypertension is a condition characterized by elevated mean pressure in the pulmonary artery (PAm) and is diagnosed using invasive right heart catheterization. 1 2 3 However, measuring mean pressure alone does not fully capture the complex physiology of the pulmonary circulation, and additional parameters are needed for an accurate diagnosis. 3 4 One such parameter is the rate of pressure change, which can be described using the equations of the water hammer effect, a phenomenon observed in pump systems with rapid changes in pressure and flow. While the water hammer effect is well-established in the systemic circulation, its relevance to pulmonary hypertension is not yet clear. 5 6 7 8

The significance of the rate of pressure change (as described in the water hammer effect) in the right ventricle (RV) has already been established. For instance, the RV dP/dt measured noninvasively using transthoracic echocardiography (TTE) has been utilized as an indicator of RV contractility. 9 10 Moreover, Ameloot et al 11 demonstrated that a dP/dt more than 400 mm Hg/s was associated with improved RV function and reduced mortality.

While the pulse wave velocity, a parameter of the water hammer equations, is a well-established indicator of vascular elasticity (based on the Moens–Korteweg equation), 7 12 its correlation with dp/dt in the pulmonary circulation is yet to be established. Since the pathophysiologic basis of pulmonary hypertension is the arterial stiffening, 13 a marker of which is the pulse wave velocity, 14 15 it is expected to correlate with the right ventricular contractility.

In this study, we aim to investigate the vascular, ventricular, and clinical parameters associated with the rate of pressure change (dP/dt) in the pulmonary artery of patients with group I pulmonary arterial hypertension (PAH). Group I PAH is a specific subtype of pulmonary hypertension characterized by elevated pulmonary vascular resistance (PVR) and poor prognosis. We will also examine whether the dP/dt in the pulmonary artery has predictive value for improvements in the 6-minute walk test (6MWT) and reduction in the N-terminal pro-B-type natriuretic peptide (NT-proBNP) in these patients.

By elucidating the relationship between dP/dt and various parameters in group I PAH patients, this study may contribute to a better understanding of the pathophysiology of pulmonary hypertension and improve clinical management of this challenging condition.

## Methods

The data for this study were collected between December 2013 and May 2014. Patients were evaluated at their first presentation and at least 3 months after their PAH diagnosis (average follow-up time was 17 ± 2.3 weeks).

Inclusion criteria for this study were as follows:

- age over 18 years,
- no previous treatment with PAH medication,
- diagnosis of PAH (clinical group 1) based on right-heart catheterization and TTE at the examination center,
- dyspnea at exercise (clinical stage II) or dyspnea limiting the exercise (clinical stage III).

Exclusion criteria were as follows:

- age under 18 years,
- previous treatment with PAH medication,
- other forms of pulmonary hypertension (clinical groups 2, 3, 4, 5), and
- dyspnea in the WHO functional stages I (no dyspnea) and IV (dyspnea at rest).

During the first examination, blood tests were performed (including NT-proBNP), as well as an electrocardiogram and a 6-MWT. When possible, cardiopulmonary exercise testing (CPET) and TTE were also conducted. Tricuspid annular plane systolic excursion (TAPSE), RV fractional area change (RV-FAC), and systolic pulmonary arterial pressure were measured using the maximum speed of the tricuspid valve and the Bernoulli equation. PAH diagnosis, differential diagnosis, and PAH therapy initiation followed the European Society of Cardiology (ESC) guidelines of 2014 ( Table S3 of Supplementary material [available in the online version]). Right-heart catheterization was used to determine the time of diagnosis of pulmonary hypertension. Patients in clinical group 1 received specific PAH therapy. For responders to the vasoreactivity test, treatment with nifedipine was initiated with a target dose of 120 mg/day; if there was intolerance, another PAH medication was prescribed.

Additionally, the following parameters were calculated: pulmonary artery compliance (CPA) and PVR. CPA was calculated using the formula C = SV/PP, where PP is the pulse pressure (systolic pressure minus diastolic pressure) and SV is stroke volume. PVR was calculated using the formula PVR = (PAm – PC)/CO, where PC is the pulmonary capillary wedge pressure, and CO is the cardiac output. The RC time was calculated using the formula τ = CPA * PVR, where *τ* is the time constancy (i.e., the time needed for the diastolic pressure to sink 37%). Arterial elastance (Ea) was calculated using the formula Ea = PVR * HF, where HF is the heart frequency. All the above measurements were performed using the pressure curves of the pulmonary artery and the RV of the right heart catheterization.

At the follow-up examination, a new 6-MWT was performed, and the NT-proBNP was measured again. The study protocol was approved by the local ethics committee, and all patients provided written informed consent.

## Measurement of dP/dt Mean PA

To calculate the dP/dt mean PA, we used the PA pressure curve obtained from the diagnostic right-heart catheterization. The ΔP/Δt ratio was calculated by dividing the pulse pressure by the time interval Δt, which is the time between PAmax and PAmin. This can be expressed as:

ΔP/Δt = (Pmax – Pmin) / Δt ( **I** )

where Pmax is the systolic pulmonary artery pressure, Pmin is the diastolic pulmonary artery pressure, and Δt is the time interval between PAmax and PAmin.

**Fig. 1:**
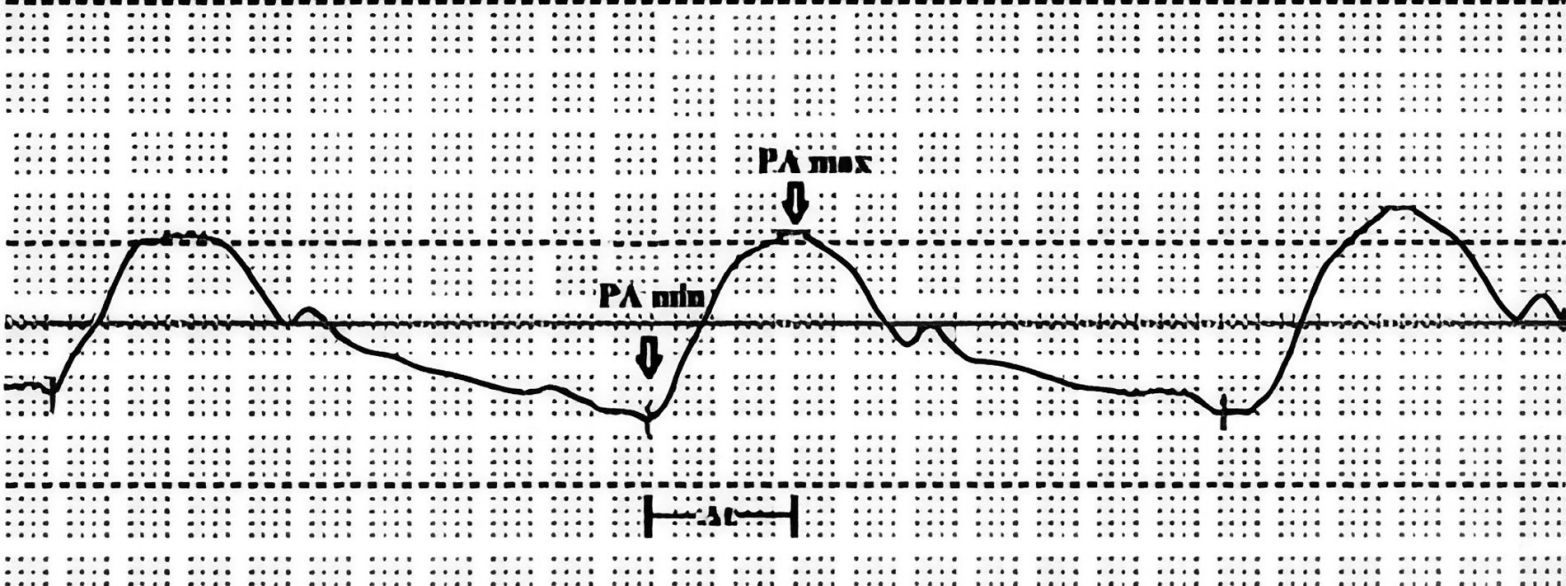
Graphical representation of a calculation of the dP/dt mean pulmonary artery (PA) from a right heart catheterization pressure curve.

Fig. 1 illustrates this measurement. If we define Δt = n*dt, where dt is the time interval between two adjacent data points in the pressure curve, then ΔP/Δt can be expressed as:

ΔP/Δt = ((P1–Pmin) + (P2–P1) + ... + (Pn-1–Pn-2) + (Pmax – Pn-1)) / (n *dt) = ΣdP / (n* dt) = (Σ dP/dt) / n = dP/dt mean

We manually measured three beats in patients in sinus rhythm and five beats in patients with atrial fibrillation from the pressure curve of the pulmonary artery. We excluded deep inspirations and expirations and used the average value of the measurements in this study.

Similarly, the dP/dt mean RV can be calculated using the equation:

dP/dt mean RV = ((RVmax – RVmin)) / Δt ( **II** )

### Statistical Analysis

Statistical analysis was conducted using SPSS 23.0G for Windows (SPSS = Statistical Package for the Social Sciences). Descriptive statistics were calculated, including measures of central tendency (mean and median), measures of dispersion (standard deviation and standard error), and range (minimum and maximum). Correlation coefficients (Pearson correlation coefficient) and regression coefficients (Beta) were used to examine the relationship between continuous variables. Multiple linear regression analyses were performed to investigate correlations with several other parameters and test for significance, with a significance level of *R* ^2^ more than 0.5 and *p* less than 0.05. The level of *R* ^2^ more than 0.5 was selected as the minimum value of significance for a linear regression model. A multivariate analysis was conducted to determine the independent influence of dP/dt mean PA, with a significance level of *p* less than 0.05. Receiver operating characteristic (ROC) curves were generated to examine the relevance of various factors in treating patients with PAH, with an acceptable discrimination set at an area under the curve (AUC) over 0.7. Cutoff values were calculated using optimal sensitivity and specificity, and odds ratios (OR) were also calculated. The study was approved by the ethics commission of the North Rhine Medical Association, and written consent was waived for the use of anonymized, retrospective data.

## Results

### Patient Population and Follow-Up

The study population was categorized into different clinical groups according to the clinical classification suggested by the guidelines of PAH. Statistical analysis was conducted only for the 142 patients in clinical group 1( Table S1 of supplementary material [available in the online version]). No deaths were recorded during the follow-up period of approximately 17 ± 2.3 weeks. Details regarding comorbidities and medication for each patient can be found in Tables S2 and S3 of the supplementary material [available in the online version], while further classification of group 1 patients can be found in Table S4 (available in the online version).

### Bivariate and Multivariate Analysis of Vascular Properties

**Fig. 2:**
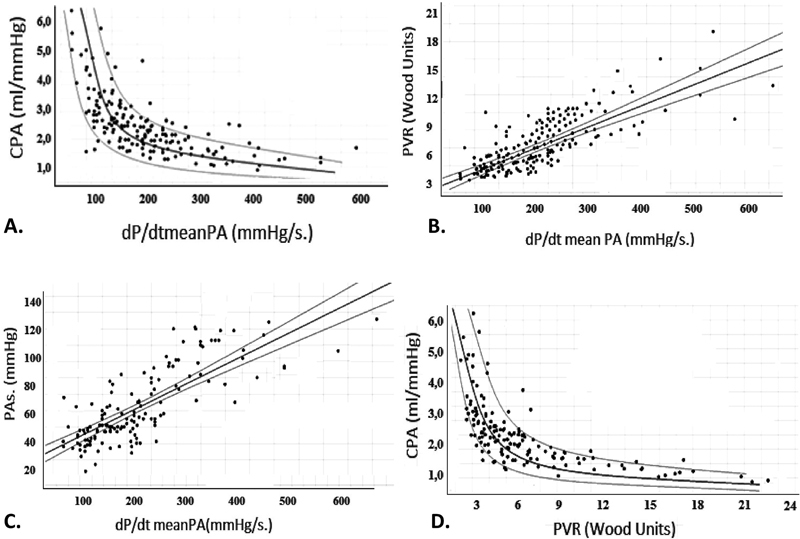
Graphical representation of the correlation of the dP/dt mean PA (X Axis in mm Hg) and the ( **A** ) CPA (Y Axis in mL/mm Hg), ( **B** ) the PVR (Y axis in Wood Units) and the ( **C** ) PAs (Y axis in mm Hg). N = 142 patients in all regressions. **D** is graphical representation of the relationship of PVR (Wood Units) and CPA (Y axis in mL/mm Hg). *p* -Values in all cases < 0.001, *R* ^2^ >0.5. CPA, pulmonary artery compliance; PA, pulmonary artery; PVR, pulmonary vascular resistance.

Regression analysis revealed a statistically significant correlation between dP/dt mean PA and systolic pressure of the pulmonary artery (PAs) ( *r* +0.75, *R* ^2^ = 0.56, *p* < 0.001), PVR ( *r =* +0.71, *R* ^2^ = 0.51, *p* < 0.001), and capillary pulmonary artery (CPA) ( *r* = −0.69, *R* ^2^ = 0.5, *p* < 0.001). The bivariate analysis of vascular parameters and dP/dt mean PA is presented in Table S5 of the supplementary material (available in the online version), while the significant relationships are illustrated in Fig. 2 .

In the multivariate analysis, the statistical significance between dP/dt mean PA and PAs and PVR were confirmed (both p < 0.05, Table S6 of the supplementary material [available in the online version]).

### Bivariate and Multivariate Analysis of RV Properties

**Fig. 3:**
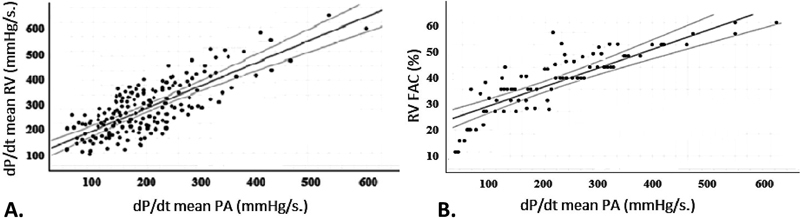
Graphical representation of the correlation of dP/dt mean PA (X Axis in mm Hg) and ( **A** ) of the dP/dt mean RV (Y Axis in mm Hg/s) and ( **B** ) RV-FAC (Y Axis in %). *p* -Values in all cases < 0.001, *R* ^2^ >0.5. PA, pulmonary artery; RV-FAC, right ventricular fractional area change.

Regression analysis revealed a statistically significant correlation between dP/dt mean PA and dP/dt mean RV ( *r* = +0.73, *R* ^2^ = 0.53, *p* < 0.001) and RV-FAC ( *r* = +0.72, *R* ^2^ = 0.51, *p* < 0.001) Fig. 3 . The results of the bivariate analysis of RV properties and dP/dt mean PA can be found in Table S7 of the supplementary material (available in the online version), while the multivariate regression analysis confirmed the statistical significance ( *p* <0.001) and is presented in Table S8 of the supplementary material (available in the online version).

### Correlations between dP/dt mean PA and Clinical Parameters

The correlation between dP/dt mean PA and various clinical parameters, including age, weight, clinical level of dyspnea, oxygen uptake and end-expiratory carbon dioxide partial pressure of cardiopulmonary exercise testing, 6-MWT, and NT-proBNP, was examined. However, no statistically significant correlation ( *R* ^2^ >0.5 and p <0.05) between dP/dt mean PA and any of the clinical factors was observed ( Table S9 of the supplementary material , available in the online version).

### Efficacy of PAH Therapy

After an average of 17 ± 2.3 weeks of PAH medication, the patients demonstrated an improvement in the 6-MWT (35 m on average) and a reduction in NT-proBNP (79 ng/L on average). A combination of both was observed in 59 patients (51.3%).

### ROC and OR Analysis

**Fig. 4:**
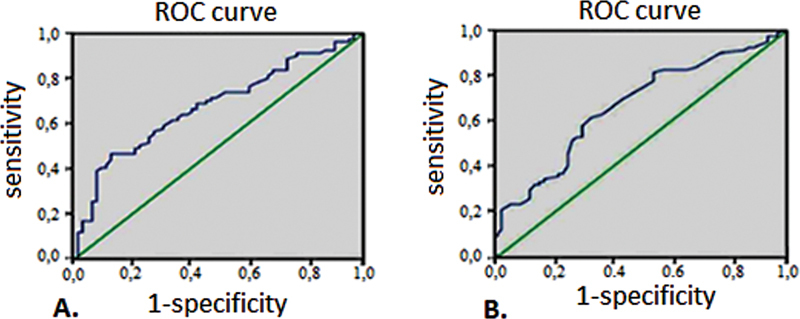
Results of the ROC analysis in respect of the combination endpoint (simultaneous improvement of the 6MWT and reduction of the NT-proBNP). ( **A** ) dP/dt mean PA, ( **B** ) dP/dt mean RV. AUC in ( **A** ) 0.73 in ( **B** ) 0.72. 6MWT, 6-minute walk test; AUC, area under the curve; PA, pulmonary artery; NT-proBNP, N-terminal-probrain natriuretic peptide; ROC, receiver operating characteristic.

The influence of hemodynamic parameters on the probability of improvement or worsening of the 6-MWT (increase or reduction), the probability of improvement or worsening of NT-proBNP (increase or decrease), and the probability of a combination of both (simultaneous increase of NT-proBNP and increase in the distance of 6-MWT, or none) was examined using ROC curves ( Tables S10 – S12 of the supplementary material , available in the online version). The highest AUC in the 6-MWT ROC analysis was 0.69 for PVR (not significant), while in the NT-proBNP analysis, it was 0.68 (also not significant). An AUC more than 0.7 had dP/dt mean PA and dP/dt mean PV in the analysis regarding the combination endpoint (presented in Fig. 4 ).

A bivariate and multivariate OR analysis was performed for those parameters with an AUC above 0.5.A statistical significance could not be confirmed ( Tables S13 and S14 of supplementary material , available in the online version).

## Discussion

In this study, the mean dP/dt of the pulmonary artery exhibited a positive correlation with several parameters, including PAs, PVR, dP/dt of the RV, and RV-FAC. Among these parameters, dP/dt of the pulmonary artery was found to be the most accurate predictor of the probability of improvement in the 6-MWT and the NT-proBNP levels following initiation of a specific medication for PAH.

It is worth noting that this study population only included patients with PAH belonging to group 1, for whom the specific medication had shown efficacy. 3 16 17 18 19 20 21 22 23 Additionally, patients with dyspnea during exercise (but not at rest) were included. The characteristics of these patients did not conform to the typical anthropometric profile of PAH patients (i.e., young females with no comorbidities). Instead, they were representative of the data collected from international registries. 24

All patients received the specific medication for PAH, and the majority showed improvement in the 6-MWT, consistent with findings from previous clinical studies 17 22 23 . However, the improvement did not meet the treatment goal set by the ESC guidelines of walking a distance of 440 meters ^3^ . Instead, a minimal important difference of approximately 30 meters was observed, as suggested by Mathai et al. 25

The data for NT-proBNP levels were also broader, with previous clinical studies showing a statistically significant reduction ranging from 13 to 60%. 16 17 18 19 20 21 22 23 In this study, a reduction of 26.7% was observed.

In this study, significant correlations were found between dP/dt mean PA and various vascular properties, including PVR, dP/dt mean RV, and RV-FAC. This is the first study to directly examine the relationship between dP/dt mean PA and PVR, 26 and the results were consistent with previous research linking both parameters to pulse wave velocity. Hubbart et al 27 previously demonstrated a direct correlation between vascular resistance and dP/dt but only in the systemic circulation. A direct correlation of PVR and pulse wave velocity as markers of arterial stiffness and increased afterload was not evaluated in this study, but rather taken for granted.

The correlation between dP/dt mean PA and dP/dt mean RV is also noteworthy, as dP/dt RV is a widely used parameter to describe RV pump function and ventricular contraction reserves, and the statistical significance of the correlation with RV-FAC suggests a possible relationship with the ventriculoarterial coupling (VAC), as suggested by Guihaire et al. 28

Importantly, dP/dt mean PA was found to be independent of heart rate, with a correlation only with PVR as a measure of afterload. Preload was not tested for statistical significance.

However, when evaluating the efficacy of PAH therapy using 6-MWT and NT-proBNP as clinical and laboratory endpoints, respectively, dP/dt mean PA was found to have no significant correlation with either the absolute numerical value or the OR analysis. Statistically significant AUC values in the ROC curve analysis could be found only for the parameters dP/dt mean PA and dP/dt mean RV, and only for analysis with the combination endpoint. The higher the dP/dt mean PA and the dP/dt mean RV are, the more likely it is that a simultaneous improvement of the 6-MWT and the NT-proBNPs occurs.

The results obtained from the study may appear contradictory at first glance, but upon closer examination, they reveal an underlying pattern. A higher dP/dt mean PA value indicates that patients have increased afterload (higher PVR) but at the same time have better RV pump function (higher dP/dt mean RV) and VAC (high RV-FAC). This could explain the higher probability of an improvement after therapy in patients with a higher dP/dt mean PA value. Conversely, a low dP/dt mean PA value is indicative of patients with advanced right heart failure, as it is unlikely for them to have a low afterload due to the essential prerequisite for the diagnosis of PAH. Therefore, patients with a low dP/dt mean PA could be treated earlier with a combination therapy to observe a clinical improvement to specific PAH medication, particularly considering that the majority of patients in this study were on monotherapy.

It is important to note that the diagnosis of PAH in this study was based on the previously recommended cutoff value of 25 mm Hg, as outlined in the earlier guidelines, 3 rather than the more recent cutoff value of 20 mm Hg. 29 Consequently, patients with a mean pressure between 20 and 25 mm Hg may not have received treatment, which could not be altered due to the retrospective design of the study.

One of the limitations of the study is that it was retrospective and right-heart catheterization was performed only once with fluid catheters, and a simultaneous presentation of the changes to the pressure and volume was not documented. Additionally, the parameter dP/dt mean PA does not account for the geometrical factors of either the RV or the pulmonary artery. The study also found no direct clinical correlation between dP/dt mean PA and other echocardiographically determined parameters except RV-FAC, nor with the clinical CPET data or with the 6-MWT or NT-proBNP, except for the combination endpoint where statistically significant AUC values were determined for dP/dt mean PA and dP/dt mean RV. The OR, however, was not relevant for the same endpoint.

In summary, although the results may seem contradictory at first, closer examination reveals a pattern that could be useful in the early diagnosis and treatment of PAH. However, the retrospective nature of the study and limitations of the parameter dP/dt mean PA should be taken into account when interpreting the results.

## Conclusion

The study demonstrated a significant correlation between dP/dt mean PA and PAs, PVR, dP/dt mean RV, and RV-FAC. Additionally, the parameter had the highest significance in predicting simultaneous improvements in 6-MWT and NT-proBNP, with an AUC of 0.73 in the ROC curve analysis. These results suggest that dP/dt mean PA is a potential indicator of elevated afterload and preserved ventricular contractility. However, while it correlates with changes in 6MWT and NT-proBNP, the study did not establish a clear prognostic role for the parameter. It is important to note that the study had limitations, including a small sample size and retrospective design.
