# Supplementary material for: Importance of the Mean Rate of Pressure Change of the Pulmonary Artery (dP/dt mean PA) in Patients with Pulmonary Arterial Hypertension
Source: Avicenna J Med. 2023 Jun 20;13(2):104–10. doi: 10.1055/s-0043-1769932 (PMC10332940; doi:10.1055/s-0043-1769932)
Supplement: Supplementary file 1 — Supplementary Material [file 10-1055-s-0043-1769932-s220121.pdf]

Supplementary Table S1 Differentiation of the study cohort, according to the Nice classification

Classification	Patient number
Nice group 1	142
Nice group 2	25
Nice group 3	21
Nice group 4	27
Nice group 5	5

n= 220 patients.

Supplementary Table S2 The most frequent comorbidities of patients in Nice group 1 (*n* = 142 patients)

Disease	Patient number	Percentage (%)
Arterial hypertension	96	67.6
Atrial fibrillation	37	26.1
Diabetes mellitus II	31	21.8
Depression	26	18.3
Hyperlipidemia	20	14.1
Stroke	13	9.2
Knee TEP/Hip TEP	13	9.2
Tumor history	11	7.8
Osteoporosis	8	5.6

Abbreviation: TEP,—

Supplementary Table S3 Patient cohort's medication

Medication	Patient number	Percentage (%)
Specific PAH medication	142	100
- PDE-5 inhibitor	95	66.9
- ET receptor antagonists	47	33.0
- Prostanoids	1	0.7
- Calcium antagonists	1	0.7
Diuretics	109	76.8
ACE inhibitor/AT2 blocker	64	45.1
(N)OAKs	37	26.0
Immunosuppressants	34	24.0
Aldosterone antagonists	32	22.5
Betablocker	32	22.5
Antidiabetic therapy	31	22.0
Oral antidiabetics	26	11.2
Insulin	9	6.3
Allopurinol	29	20.4

Supplementary Table S3 (*Continued*)

Medication	Patient number	Percentage (%)
Antidepressants	26	8.5
Morphine	14	9.9
Central antihypertensives	10	11.2
Digitalis	8	5.6
Iron replacement	7	4.9

Abbreviations: ACE,—; ET,—; PAH, pulmonary arterial hypertension; PDE-5,—.

Supplementary Table S4 Further classification of patients with group 1 pulmonary hypertension

Etiology of PAH	Patients	Percentage (%)
Idiopathic PAH	98	69.0
Associated PAH with:	42	29.6
- Collagenosis	34	24.0
- Portal hypertension	4	2.8
- Congenital systemic pulmonary shunts	3	2.1
- HIV infection	1	0.7
Drug and toxin induced	1	0.7
PVOD	1	0.7

Abbreviations: PAH, pulmonary arterial hypertension; PVOD, pulmonary veno-occlusive disease; HIV, human immunodeficiency virus.

Supplementary Table S5 Bivariate analysis of the pulmonary artery vascular parameters with dP/dt mean PA

Parameter	MW ± SW	*r*	R^2	*p*-Value
PAs (mm Hg)	61.8 ± 22.9	0.75	0.56	<0.001
PVR (Wood units)	6 ± 3.6	0.71	0.51	<0.001
CPA (mL/mm Hg)	2 ± 1.1	−0.69	0.50	<0.001
Ea (mm Hg/L)	7.6 ± 5.1	0.65	0.42	<0.001
PAm(mm Hg)	38.7 ± 12.9	0.64	0.40	<0.001
PAd (mm Hg)	22.9 ± 9.7	0.41	0.17	<0.001
PA-sat. (%)	61.7 ± 8.3	0.28	0.08	0.001
RC-time (seconds)	9.6 ± 3.1	0.13	0.02	0.11
Heart rate	74 ± 14	0.06	0.004	<0.001

Abbreviations: CPA, pulmonary artery compliance; MW,—; PA, pulmonary artery; PAd, diastolic pulmonary pressure; Pas, systolic pulmonary pressure; PA-Sat, oxygen saturation in PA; PVR, pulmonary vascular resistance; SW,—.
n= 142 patients in each regression analysis. The *r*-, R^2-, and *p*-values result from the regression analysis with dP/dt mean PA. Significant correlations are shown in red.

 © 2023. The Author(s).

Supplementary Table S6 Multivariate analysis of the PA vascular parameters with dP/dt mean PA

Parameter	*p*-Value	Beta	95% lower	95% upper
PAs	**<0.001**	**4.09**	**1.87**	**6.31**
PAd	**0.005**	**–4.46**	**7.54**	**–1.37**
PVR	**0.02**	**31.29**	**4.84**	**57.74**
Ea	0.09	−0.25	−0.54	0.04
HF	0.12	1.5	−0.42	3.49
RC-time	0.56	−2.4	−0.54	0.04
PA-sat	0.67	−0.33	−1.89	1.22
PAm	0.72	−0.79	−5.12	3.56
CPA	0.98	−0.24	−25.5	25.03

Abbreviations: CPA, pulmonary artery compliance; PA, pulmonary artery; Pad, diastolic pulmonary pressure; PAs, systolic pulmonary pressure; PA-sat, oxygen saturation in PA; PVR, pulmonary vascular resistance.
The *p*-values result from the regression analysis with dP/dt mean PA. Significant influencing factors are shown in red.

Supplementary Table S7 Bivariate analysis of the right ventricular parameters and of the dP/dt mean PA

Parameter	Patients	MW ± SD	*r*	R^2	*p*-Value
dP/dt RV mean (mmHg/s)	142	254.35 ± 110.95	0.73	0.53	<0.001
RV-FAC (%)	104	35.5 ± 7.2	0.72	0.51	<0.001
RV sys. (mm Hg)	142	58.88 ± 21.41	0.6	0.35	<0.001
RV endd. (mm Hg)	142	9.04 ± 5.33	0.43	0.18	<0.001
RA-sat (%)	142	61.84 ± 8.62	0.29	0.09	<0.001
MVO_2(%)	142	61.56 ± 7.25	0.25	0.07	0.02
SV (mL)	142	68.77 ± 30.52	0.22	0.05	0.009
AO-sat (%)	142	91.36 ± 5.03	0.17	0.03	0.04
RA (mm Hg)	142	8.62 ± 5.34	0.17	0.03	0.04
TK sys.(mm Hg)	124	61.6 ± 29.1	0.14	0.02	0.11
RV d. (mm Hg)	142	4.04 ± 4.34	0.11	0.01	0.18
TAPSE (mm)	129	18.2 ± 5.65	0.02	0.01	0.86

Abbreviations: AO,—; MVO2,—; MW, minute walk; PA, pulmonary artery; RA,—; RV-FAC, right ventricular fractional area change; SD, standard deviation; SV,—; TK,—.
The *r*-, *R*-square, and *p*-values come from the regression analysis with dP/dt mean PA. Statistically significant regressions are shown in red.

 © 2023. The Author(s).

Supplementary Table S8 Multivariate analysis of the right ventricular parameters and of the dP/dt mean PA

Parameter	*p*-Values	Beta	95% lower	95% upper
dP/dt mean RV	<0.001	0.47	0.27	0.67
RV-FAC	<0.001	5.27	2.81	7.73
RV endd.	0.07	6.80	−0.59	14.2
HF	0.14	−0.85	−2.00	0.28
RA	0.16	−4.55	−10.96	1.86
RV s.	0.22	0.59	−0.36	1.53
TK sys.	0.26	−0.33	−0.90	0.25
RV d.	0.50	−1.92	−7.55	3.70
SV	0.52	−0.25	−1.00	0.51
AO-sat	0.71	−0.60	−3.81	2.61
TAPSE	0.72	−0.56	−3.60	2.48
RA-sat	0.78	0.40	−2.55	3.36
MVO_2	0.83	−0.40	−3.92	3.13

Abbreviations: AO,—; HF, heart failure; MVO2,—; MW, minute walk; PA, pulmonary artery; RA,—; RV-FAC, right ventricular fractional area change; SD, standard deviation; SV,—. ; TK,—.
The *p*-values result from the regression analysis with dP/dt mean PA. Significant influencing factors are shown in red.

Supplementary Table S9 Clinical factors and statistical correlations with dP/dt mean PA

Parameter	Patients	MW ± SD	*r*	R^2	*p*-Value
6-MWT1	115	285 ± 119	0.24	0.06	0.01
WHO level	142	2.6 ± 0.45	0.07	0.01	0.46
Weight (kg)	142	78.9 ± 18.8	0.08	0.01	0.39
Age (years)	142	68.4 ± 12.4	0.12	0.01	0.29
Height (cm)	142	166.9 ± 8.5	0.08	0.01	0.43
VO_2 max. (l/Min)	89	1.11 ± 0.4	0.06	0.004	0.57
6-MWT2	115	320 ± 116	0.05	0.003	0.58
$PETCO_2$ (mm Hg)	49	13 ± 4.1	0.05	0.002	0.74
NT-proBNP1	142	2.543 ± 6.594	0.02	0.001	0.84
NT-proBNP2	142	1.864 ± 2.948	0.01	0.001	0.92

Abbreviations: 6MWT1, 6-minute walk test1; NT-proBNP, N-terminal-probrain natriuretic peptide; PA, pulmonary artery; PAH, pulmonary arterial hypertension.
The r-, R^2, and *p*-values result from the regression analysis with dP/dt mean PA.
6MGT1 = 6-minute walk test before the initiation of a specific PAH medication,
6MGT2 = 6-minute walk test after the initiation of a specific PAH medication.
NT-proBNP1 = NT-proBNP value before the initiation of a specific PAH medication.
NT-proBNP2 = NT-proBNP value after the initiation of a specific PAH medication.
PETCO2= end tidal carbon dioxide pressure.
VO2max = maximum oxygen uptake.

 © 2023. The Author(s).

Supplementary Table S10 Results of the ROC analysis in respect of the 6 MWT

Parameter	AUC	95% lower	95% upper	Cutoff (Units)	Sensitivity	Specificity
PVR	0.69	0.59	0.79	4.3 (W.U.)	0.75	0.54
RVs	0.68	0.57	0.78	49.5 (mm Hg)	0.71	0.54
dP/dt RV	0.66	0.56	0.76	217 (mm Hg/s.)	0.69	0.54
dP/dt PA	0.66	0.56	0.76	203 (mm Hg/s.)	0.67	0.56
TKs	0.66	0.54	0.77	51.5 (mm Hg)	0.77	0.53
Ea	0.65	0.55	0.75	0.51 (mm Hg/mL)	0.67	0.54
RV-FAC	0.64	0.52	0.76	36.25 (%)	0.61	0.60
PAs.	0.64	0.53	0.74	51.5 (mm Hg)	0.71	0.56
PA m	0.63	0.52	0.73	33.5 (mm Hg)	0.65	0.56
RVd.	0.61	0.51	0.72	2.5 (mm Hg)	0.58	0.56
PA d	0.60	0.49	0.70	20.5 (mm Hg)	0.54	0.51
RVendd.	0.60	0.5	0.71	8.5 (mm Hg)	0.49	0.66
RA	0.56	0.45	0.67	7.5 (mm Hg)	0.60	0.46
Height	0.54	0.43	0.65	167.5 (cm)	0.57	0.61
RC time	0.52	0.40	0.63	9.3 (1/s)	0.52	0.61
WHO Kl.	0.51	0.40	0.62	2.75	0.53	0.56
HF	0.51	0.40	0.62	73.5 (beats/Min.)	0.54	0.66
Weight	0.47	0.36	0.58			
TAPSE	0.46	0.35	0.58			
Age	0.45	0.34	0.56			
VO_2	0.44	0.30	0.59			
AO-sat.	0.44	0.32	0.54			
$PETCO_2$	0.43	0.29	0.62			
PC	0.43	0.32	0.54			
PA-sat.	0.42	0.31	0.53			
RA-sat.	0.39	0.28	0.51			
SV	0.38	0.27	0.48			
VO_2 soll	0.37	0.23	0.51			
MvO_2	0.37	0.27	0.49			
CPA	0.34	0.24	0.44			

Abbreviations: AUC, area under the curve; CPA, pulmonary artery compliance; HF, heart failure; PA, pulmonary artery; PVR, pulmonary vascular resistance; ROC, receiver operating characteristic; RV-FAC, right ventricular fractional area change; W.U., Wood Unit.
95% lower and 95% upper = lower and upper thresholds of the 95% confidence interval. Units in parentheses. *n* = 115 patients.

 © 2023. The Author(s).

Supplementary Table S11 Results of the ROC analysis in respect of the NT-proBNP

Parameter	AUC	95% lower	95% upper	Cutoff (units)	Sensitivity	Specificity
dP/dt PA	0.68	0.59	0.77	209 (mm Hg/s.)	0.62	0.66
RV s.	0.67	0.58	0.76	49.5 (mm Hg)	0.69	0.57
dP/dt RV	0.66	0.57	0.75	213 (mm Hg/s.)	0.64	0.54
RV-FAC	0.65	0.54	0.75	36.25 (%)	0.62	0.65
PVR	0.65	0.56	0.74	4.7 (W.U.)	0.62	0.56
PA m	0.64	0.55	0.73	32.5 (mm Hg)	0.63	0.56
PA s	0.64	0.55	0.73	52.5 (mm Hg)	0.63	0.59
Ea	0.61	0.52	0.71	0.51 (mm Hg/mL)	0.64	0.54
PA d	0.60	0.50	0.69	20.5 (mm Hg)	0.54	0.62
HF	0.55	0.46	0.65	71.5 (beats/Min.)	0.59	0.47
TK s	0.54	0.44	0.65	55.5 (mm Hg)	0.57	0.54
RC time	0.54	0.44	0.63	8.8 (1/s)	0.64	0.45
RA	0.52	0.43	0.62	7.5 (mm Hg)	0.59	0.43
Height	0.52	0.42	0.61	163.5 (cm)	0.64	0.38
TAPSE	0.51	0.41	0.61	16.5 (mm)	0.53	0.43
RV endd.	0.49	0.39	0.58			
Weight	0.47	0.37	0.56			
WHO Kl.	0.46	0.36	0.56			
VO_2	0.45	0.33	0.58			
SV	0.45	0.36	0.55			
RV d.	0.44	0.34	0.54			
AO-sat.	0.41	0.32	0.51			
$PETCO_2$	0.41	0.25	0.58			
PC	0.40	0.29	0.50			
Alter	0.39	0.30	0.48			
CPA	0.39	0.29	0.48			
MvO_2	0.39	0.30	0.48			
VO_2 soll	0.38	0.26	0.49			
RA-sat.	0.37	0.27	0.46			
PA-sat.	0.34	0.25	0.43			

Abbreviations: AUC, area under the curve; CPA, pulmonary artery compliance; HF, heart failure; PA, pulmonary artery; NT-proBNP, N-terminal-probrain natriuretic peptide; PVR, pulmonary vascular resistance; ROC, receiver operating characteristic; RV-FAC, right ventricular fractional area change; W.U., Wood Unit.
95% lower and 95% upper = 95% confidence interval. Units in parentheses. n = 142 patients.

 © 2023. The Author(s).

Supplementary Table S12 Results of the ROC analysis in respect of the combination endpoint

Parameter	AUC	95% lower	95% upper	Cutoff (units)	Sensitivity	Specificity
dP/dt PA	0.73	0.63	0.82	204 (mm Hg/s.)	0.76	0.56
dP/dt RV	0.72	0.62	0.81	228 (mm Hg/s.)	0.73	0.59
RV s.	0.70	0.61	0.80	50.5 (mm Hg)	0.74	0.57
PVR	0.68	0.58	0.78	5.1 (W.U.)	0.65	0.57
PA s.	0.68	0.58	0.78	52.5 (mm Hg)	0.76	0.52
RV-FAC	0.68	0.57	0.79	36.25 (%)	0.71	0.62
PA m.	0.67	0.57	0.77	35.5 (mm Hg)	0.61	0.59
Ea	0.65	0.55	0.76	0.59 (mm Hg/mL)	0.63	0.41
PA d.	0.62	0.51	0.72	21.5 (mm Hg)	0.57	0.62
TKs.	0.59	0.47	0.70	59.5 (mmHg)	0.57	0.57
RA	0.56	0.45	0.66	7.5 (mm Hg)	0.63	0.48
HF	0.56	0.45	0.67	72.5 (beats/min.)	0.59	0.57
RV d.	0.55	0.44	0.65	3.5 (mm Hg)	0.48	0.64
RV endd.	0.55	0.45	0.66	8.5 (mm Hg)	0.48	0.61
Height	0.53	0.42	0.63	167.5 (cm)	0.52	0.51
TAPSE	0.53	0.42	0.64	17.5 (mm)	0.49	0.51
RC time	0.52	0.41	0.62	9.3 (1/s)	0.54	0.56
VO_2	0.49	0.35	0.63			
WHO Kl.	0.48	0.38	0.59			
Weight	0.47	0.34	0.55			
$PETCO_2$	0.47	0.29	0.66			
Age	0.42	0.31	0.53			
SV	0.42	0.31	0.52			
PC	0.41	0.31	0.52			
MvO_2	0.38	0.27	0.48			
PA-sat.	0.38	0.27	0.48			
AO-sat.	0.39	0.29	0.49			
CPA	0.39	0.23	0.43			
RA-sat.	0.37	0.27	0.48			
VO_2 soll	0.36	0.23	0.49			

Abbreviations: AUC, area under the curve; CPA, pulmonary artery compliance; HF, heart failure; PA, pulmonary artery; PVR, pulmonary vascular resistance; ROC, receiver operating characteristic; RV-FAC, right ventricular fractional area change; W.U., Wood Unit.
95% lower and 95% upper = 95% confidence interval. Units in parentheses. $n = 115$ patients.

 © 2023. The Author(s).

Supplementary Table S13 Bivariate OR of the various hemodynamic parameters with the combination endpoint

Combination endpoint			
Parameter	OR	95% lower	95% upper
Ea	9.47	2.23	39.4
PVR	1.3	1.11	1.47
RV-FAC	1.1	1.02	1.18
RVendd	1.08	0.97	1.21
RA	1.06	0.97	1.16
PAd	1.05	1	1.09
PAm	1.05	1.02	1.09
RV s	1.04	1.02	1.06
TAPSE	1.04	0.97	1.11
PAs	1.03	1.01	1.05
HF	1.02	0.99	1.05
RVd	1.02	0.94	1.1
TKs	1.02	1	1.04
RC time	1.02	0.9	1.16
Height	1.01	0.97	1.05
dP/dt mean PA	1.01	1.01	1.02
dP/dt mean RV	1.01	1	1.01

Abbreviations: HF, heart failure; OR, odds ratio; PA, pulmonary artery; PVR, pulmonary vascular resistance; RV-FAC, right ventricular fractional area change.
95% lower and 95% upper = 95% confidence interval. Units in parentheses. $n = 115$ patients.

Supplementary Table S14 Multivariate OR of the various hemodynamic parameters with the combination endpoint

Combination endpoint			
Parameter	OR	95% lower	95% upper
Ea	2.47	0.35	7.44
RVd	1.3	0.99	1.71
RC time	1.13	0.59	2.17
Height	1.08	0.96	1.2
RV s	1.06	0.94	1.18
PAd	1.06	0.83	1.36
TAPSE	1.05	0.89	1.22
PVR	1.04	0.35	3.05
RA	1.04	0.82	1.32
HF	1.02	0.94	1.12
TKs	1.01	0.98	1.04
dP/dt mean PA	1.01	1	1.04
dP/dt mean RV	1.01	1	1.02
RVendd	0.97	0.68	1.3
PAs	0.97	0.77	1.22
RV-FAC	0.92	0.76	1.01
PAm	0.88	0.61	1.27

Abbreviations: HF, heart failure; OR, odds ratio; PA, pulmonary artery; PVR, pulmonary vascular resistance; RV-FAC, right ventricular fractional area change.
95% lower and 95% upper = 95% confidence interval. Units in parentheses. $n = 115$ patients.

 © 2023. The Author(s).
